# Effective use of Item Analysis to improve the Reliability and Validity of Undergraduate Medical Examinations: Evaluating the same exam over many years: a different approach

**DOI:** 10.12669/pjms.41.3.10693

**Published:** 2025-03

**Authors:** Nadeem Alam Zubairi, Turki Saad AlAhmadi, Mohamed Hesham Ibrahim, Moustafa Abdelaal Hegazi, Fahad Ussif Gadi

**Affiliations:** 1 Nadeem Alam Zubairi, Department of Pediatrics, Faculty of Medicine, King Abdulaziz University, Rabigh, Saudi Arabia; 2 Turki Saad AlAhmadi, Department of Pediatrics, Faculty of Medicine, King Abdulaziz University, Rabigh, Saudi Arabia; 3 Mohamed Hesham Ibrahim, Department of Pediatrics, Faculty of Medicine, King Abdulaziz University, Rabigh, Saudi Arabia; 4 Moustafa Abdelaal Hegazi, Department of Pediatrics, Faculty of Medicine, King Abdulaziz University, Rabigh, Saudi Arabia; 5 Fahad Ussif Gadi, Department of Pediatrics, Faculty of Medicine, King Abdulaziz University, Rabigh, Saudi Arabia

**Keywords:** Difficulty Index, Discrimination Index, Distractor Efficiency, Exam Reliability, Item Analysis

## Abstract

**Objective:**

MCQ exams are part of end-module assessments in undergraduate medical institutions. Item Analysis (IA) is the best tool to check their reliability and validity. It provides the Reliability Coefficient KR20, Difficulty Index (DI), Discrimination Index (DISC), and Distractor Efficiency (DE). Almost all research papers on IA are based on single exam analysis. We examined the IA of multiple exams of the same module, taken in four years. We aimed to explore the required consistency over the years and the effectiveness of IA-based post-exam measures.

**Methodology:**

Item Analysis of eight final MCQ exams of the Pediatric module from 2020-21 to 2023-24, at the Faculty of Medicine in Rabigh, King Abdulaziz University, Saudi Arabia, were included in the study.

**Results:**

All exams had KR20 of 90 and above indicating excellent reliability. Difficulty levels were consistent except for a single year. Discriminative ability was maintained over the years. Only 28 out of 800 MCQs had a negative DISC. All exams maintained good DE. Only 15 MCQs over four years had zero DE. The practice of reviewing all Non-Functional Distractors yielded a gradual improvement in exam quality

**Conclusion:**

Besides the IA of individual exams, it is also recommended that IA of the same exam be evaluated over 4-5 years to see consistency and trends towards improvement. It helps in improving the reliability and validity by addressing deficiencies and deviations from the recommended standards

## INTRODUCTION

Assessment is an essential element of the learning process. Assessments have to be accurate and should be validated. MCQ exams at the end of modules are a regular component in all undergraduate medical institutions. Since the last decade, more and more medical colleges have adopted Item Analysis (IA) as a validation tool for MCQ examinations.^1^ It gives constructive feedback related to the quality of items and helps in improving the effectiveness of these exams besides providing reliability and validity. Post-exam IA assists in retaining good MCQs, modifying those with minor issues, and removing those with major defects from the question bank. Feedback from IA may also give clues for modifying instruction methods.^2^

Almost all the research papers on IA in the past have focused on IA of a single exam. Very few have included more than one exam in their study addressing a single factor.^3^ One study from Bahrain examined IA of multiple years but with different objectives.^4^ In this study, authors have explored the IA of 08 exams taken in the last 04 years for the same module. The study aimed to evaluate the same exam over the years for consistency in different aspects of an MCQ exam. It intended to see the related trends and patterns emerging due to post-exam measures based on IA and how these comparisons can be employed to improve future exams.

## METHOD

The study was conducted at the Faculty of Medicine in Rabigh, King Abdulaziz University, Kingdom of Saudi Arabia. Item Analysis of final MCQ exams of the Pediatric module from 2020-21 to 2023-24 were included in the study. Thus, a total of 08 exams were compared, as two batches are trained and examined each year, one in each semester. Each exam had 100 Type “A” MCQs with one correct and three incorrect options (distractors) for each MCQ. The data obtained from the item analysis were categorized, tabulated in Excel, and analysed by SPSS V27.

### Ethical Approval:

Ethical Committee’s approval was taken for the research (Ref 24022 dated 10/10/2024)

Reliability Coefficient KR20 and other descriptive parameters related to students’ scores were explored. The difficulty level of all exams was checked by measuring the Difficulty Index (DI), using response frequencies for each question, and then grouping these into subsets. In previous studies related to IA, researchers have made variable numbers of groups, from 03 to as many as 10 groups, for each exam to show ease or difficulty.^5^-^7^ Authors selected four subgroups, to have a balanced depiction of DI (Table-III), considering the number of MCQs and the relatively small number of students appearing in our exams (average 36) influencing the number of subgroups and their ranges.

**Table-I:** Index of Discrimination (DISC)

S.No.	Levels of Discrimination	Description
1	0.40 & above	Very good items
2	0.30–0.39	Reasonably good items
3	0.20–0.29	Marginal items (Subjected to improvement)
4	0.19 Or less	Poor items (to be rejected or improved by revision)

**Table-II:** Comparison of reliability and students’ scores from 2020-21 to 2023-24

Year	Module	Reliability Coefficient (KR20):	Total Possible Points	Mean Score	Median Score	Range of Scores	Maximum Score	Minimum Score	Students
2020-21	1^st^ Semester	0.94	100	71.78	79	53	92	39	27
2020-21	2^nd^ Semester	0.90	100	65.65	64	58	88	30	37
2021-22	1^st^ Semester	0.96	100	58.53	63.50	54	87	33	38
2021-22	2^nd^ Semester	0.92	100	75.05	76	46	93	47	37
2022-23	1^st^ Semester	0.95	100	66.25	64.5	54	94	40	41
2022-23	2^nd^ Semester	0.94	100	69.56	71	58	95	37	37
2023-24	1^st^ Semester	0.91	100	61.03	62	48	86	38	33
2023-24	2^nd^ Semester	0.92	100	62.51	64	49	89	40	37

**Table-III:** Difficulty Index of all exams

Year	Module	Percentage of correct answers			
		Below 15% (Very difficult)	15-35% (Difficult)	35-85% (Average)	Above 85% (Easy)
2020-21	1^st^ Semester	2	4	72	22
2020-21	2^nd^ Semester	0	10	74	16
2021-22	1^st^ Semester	1	8	90	1
2021-22	2^nd^ Semester	1	3	59	37
2022-23	1^st^ Semester	2	10	74	14
2022-23	2^nd^ Semester	1	3	77	19
2023-24	1^st^ Semester	0	13	74	13
2023-24	2^nd^ Semester	1	13	74	12

Point biserial correlation was used for the discrimination coefficient as it covers the responses from all students instead of 54% and correlates better with the overall student score.^8^ The term Discrimination Index (DISC) however is used for ease of understanding. Authors adopted the ranges proposed by Velou MS and others,^9^,^10^ as shown in Table-I.

For Distractor Efficiency (DE), the number of Non-Functional Distractors (NFDs) in each MCQ and of the whole exam was calculated (an NFD is a distractor that is chosen by less than 5% of the respondents). This provided us with the number of MCQs having 0,1,2 or 3 NFDs, representing 100%, 66%,33%, and 0% DE respectively

All these parameters were critically analyzed to check consistency over the eight exams in the last 04 years. Trends and patterns were also explored, considering the post-exam practices being adopted at the concerned department. Inferences were drawn, for better utility of previous exams’ analysis for future improvement.

## RESULTS

The reliability Coefficient (KR20) and other score-related parameters of last 04 years exams are shown in Table-II.

Difficulty Indices of all eight exams, year and semester-wise, are depicted in Table-III. Discrimination Indices of final MCQ exams of the last 04 years are shown in Table-IV. Results of Distractor Efficiency are given in Table-V.

**Table-IV:** Index of Discrimination

Year	Module	Point Biserial					
		0.19 or less	0.20-0.29	0.30- 0.39	0.4 and above	Zero	in minus
		Poor items	Marginal items	Reasonably good	Very good items		
2020-21	1^st^ Semester	09	13	19	50	6	3
2020-21	2^nd^ Semester	16	18	24	34	1	7
2021-22	1^st^ Semester	07	07	15	69	0	2
2021-22	2^nd^ Semester	20	18	15	40	4	3
2022-23	1^st^ Semester	11	14	20	52	2	1
2022-23	2^nd^ Semester	13	14	16	52	2	3
2023-24	1^st^ Semester	16	16	21	38	2	7
2023-24	2^nd^ Semester	14	20	27	36	1	2

**Table-V:** Distractor Efficiency (DE) and Non-Functional Distractors (NFD) (100 MCQs and 300 Distractors per exam)

Year	Module	Number of MCQs with their DE				Effective distractors	Total NFD
		0 NFD (100%)	1 NFD (66%)	2 NFDs (33%)	3 NFDs (0%)		
2020-21	1^st^ Semester	43	37	15	5	218	82
2020-21	2^nd^ Semester	63	29	7	1	254	46
2021-22	1^st^ Semester	78	20	2	0	276	24
2021-22	2^nd^ Semester	44	39	12	5	222	78
2022-23	1^st^ Semester	37	44	17	2	216	84
2022-23	2^nd^ Semester	63	27	9	1	252	48
2023-24	1^st^ Semester	71	23	5	1	264	36
2023-24	2^nd^ Semester	75	22	3	0	272	28

## DISCUSSION

Item Analysis (IA) of MCQ exams has gradually become the essential tool for checking the reliability and validity of that exam. It helps the faculty for assessment of the standard of the exam including its reliability, level of difficulty, discriminative ability, and standard of constructed MCQs. These are achieved by generating Reliability Coefficient KR20 (RC), difficulty index (DI), discrimination index (DISC), and distractor efficiency (DE). This check on reliability and validity is ideally coupled with the post-exam process which also includes scrutiny of individual MCQs deviating from desirable standards in terms of extremes of difficulty or ease, poor or negative discriminative power, and having a poor or zero DE.

In contrast to most research papers concerning IA which discuss individual exams, we explored IA of 08 exams of the same style and module over 04 years as mentioned in the introduction

### Reliability:

Our study revealed that all 08 exams over the last four years maintained an excellent Reliability Coefficient KR 20 (Table-II). Minimum was 0.90 and maximum was 0.96 (Average 0.93). According to medical educationists, values of 0.8 and above for high-stakes exams are desirable and a KR-20 of 0.90 and above is optimal.^11^ Medical undergraduate institutes should explore RC KR20 of their exams for each department, covering the last 4-5 years, and address any broad variation besides reasons for scores below 0.80. Other than psychometric parameters, descriptive statistics were also explored (Table-II). These descriptive statistics also support the general uniformity of exams over four years.

### Index of Difficulty (DI):

An important psychometric parameter to consider is DI. It tells us about the level of difficulty or ease of each MCQ by calculating the number of students answering it correctly. When DI of all questions is considered, it provides an overall picture of that exam, whether it is easy, difficult, or standard. Different educationists have proposed different percentages of questions to be in the range of easiness or difficulty, however, the general agreement is that most of the questions should be moderately difficult.^6^,^12^ It was noted that the Difficulty Indices of all the semesters for years 2020-21, 2022-23, and 2023-24, achieved that and had insignificant differences between them in terms of desired ease/difficulty. The percentage of questions with average difficulty in 06 exams of these three years had a narrow range from 72-77% (average 74%). However, both exams of the year 2021-22 were markedly different from other years in terms of difficulty levels and even more different from each other (Table-III). Students found the first-semester exam as difficult. Many reasons can be associated with the difficulty of a single item, such as flaws in the writing, uncovered content material, and even a wrong key,^13^ but when an exam has too many difficult questions the causes behind include factors other than the exam itself. A student group may have inherently weaker abilities. For example, the same exam may have different difficulty levels for students in a government medical college compared to a private one.^14^ Situational effects do play a part as many experienced during and after the Covid pandemic. The subsequent 2^nd-^semester exam, however, of the same year was much easier in terms of difficulty index. Was that an unintentional consequence of having a previous difficult exam? This exam also had relatively weaker other parameters, which supports this hypothesis.

### Index of Discrimination (DISC):

All the 08 exams showed a good overall ability to discriminate between competent (high achievers) and less competent (low achievers) students. (Table-IV). This is critical for being fair with all those who have put effort and time into excelling. The range of DISC is from 1.0 to minus 1.0. A positive DISC indicates that more high achievers have correctly answered the question than low achievers. This is the desirable goal. MCQs with negative DISC reflect the opposite and are not desirable. Reasons behind questions with negative discrimination include item flaws, poor distractors, use of vague words, and questions related to controversial issues.^15^

Although DISC of more than 0.15 is accepted as evidence of item validity, the desirable values are 0.3 and above,^9^,^15^ as shown in Table-I. In our study, the average percentage of questions with DISC above 0.3 for all years was 66% (Range 55-84%). There was general uniformity in DISC over the years. It is also known that the best discriminative ability is in the questions with moderate difficulty.^16^The average of questions with moderate difficulty for all 08 exams in our study was 74.25% (Table-III), which is additional evidence that these exams were well discriminating. There were 28 MCQs, out of a total of 800 in all exams over 04 years, with negative discriminative values. All such questions are subjected to scrutiny and while all are dropped from the question bank, only those with major flaws are cancelled.

### Distractor Efficiency (DE):

Distractors are the wrong options in Type A MCQs. These are supposed to be plausible and convey a mis-concept. These should be otherwise similar in length and style. The ability of the wrong options to distract the students is called Distractor Efficiency DE.^15^ For any question to have 100% DE, it shouldn’t have any Non-Functional Distractor (NFD), and it is considered excellent and desirable (a NFD is a distractor chosen by less than 5% of the respondents). Accordingly, the DE decreases as the number of NFDs increases. MCQs with 1,2 or 3 NFDs represent 66%,33%, and 0% DE respectively. The presence of NFDs makes an MCQ easier and affects both DI and the discriminative power.^13^ The reliability of an exam is negatively affected by the presence of NFD in excess and MCQs with poor DE should be scrutinized to identify and remove item flaws.^17^

**Fig.1:**
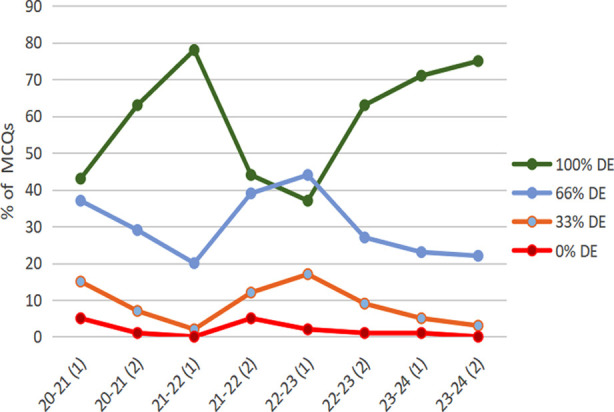
Percentages of MCQs with their Distractor Efficiencies (DE).

Studying the DF of all understudy 08 exams revealed an overall good consistency over the years as shown in Table-V and is evident in Fig.1.

**Fig.2:**
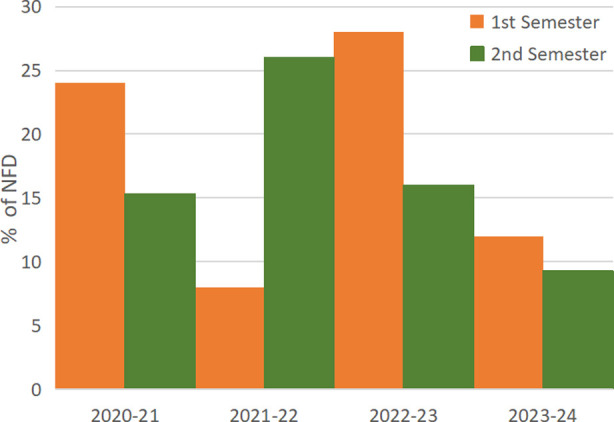
Percentages of Non-Functional Distractors (NFD).

The number of MCQs with zero DE remains persistently low. Out of 800 MCQs under consideration only 15 had zero DE. In contrast, the average number of MCQs with 100% DE in all exams is 60% (Range 37-78). A constant trend towards improvement is noticeable in the last 3 exams. This complements the trend apparent while exploring the number/percentages of NFDs over the years (Table-V and Fig.2).

Last year the two exams had 36 (12%) and 28(9%) NFDs out of 300 in each exam. This gradual improvement is secondary to the policy of letting the faculty review and replace all NFDs of that exam before that MCQ is readmitted to the question bank. NFDs are required to be replaced with more effective ones, to improve the quality of subsequent exams. This practice of refining the existing MCQs also helps the faculty when they are constructing new MCQs.

Developing a good Item bank is essential for a transparent and fair assessment. This is only possible through a regular practice of item vetting.^18^ We recommend all undergraduate medical institutions to involve the faculty members in reviewing the MCQs of their last exams through IA. Flaws should be identified and rectified. Institutions should also perceive and recognize the general pattern towards improvement or otherwise through the years. Medical education/Quality assurance units are required to supervise and work with individual departments, to secure maintenance of required standards besides ensuring gradual improvement over the years. Sharing inter-departmental experiences and regular workshops will help. All this is part of the process of safeguarding the production of competent healthcare professionals.

### Limitations:

Although the number of MCQs is sufficient, the number of students in each exam was small and a larger sample of respondents would have been more effective.

### Strength:

The study applied a different approach, utilizing IA over the years to explore consistency in KR-20, DI, DISC, and DE. All exams were from the same curriculum and module and had the same style.

## CONCLUSION

Item Analysis (IA) of every high-stakes MCQ exam is needed to check the reliability and validity. Usually, IA is confined to one specific exam by checking the reliability coefficient, difficulty index, discrimination index, and distractor efficiency. We employed a rather different approach by exploring all these for 08 exams of the same module over 04 years, checking the consistency, and evaluating the effectiveness of post-exam IA on subsequent exams by identifying related trends and patterns. It was also sought how these comparisons can be utilized, for improving future exams. We recommend that all departments in a medical college should explore the IA of their final module exams of the last 4-5 years to see the uniformity and address any inconsistencies and reasons for poor reliability or validity. It is also recommended for the Medical Education and Quality departments to supervise the process as a policy. The effectiveness of post-exam IA should be evident by the maintenance/improvement of good difficulty index, discriminating index, and distractor efficiency of subsequent exams.

### Abbreviations:

***MCQs:*** Multiple-choice questions ***IA:*** Item analysis

***OSCE:*** Objective Structured Clinical Exam ***KR-20:*** Kuder–Richardson formulas

***DI:*** Difficulty index ***DISC:*** Discrimination index

***DE:*** Distractor efficiency ***NFD:*** Non-functioning distractors

### Authors’ Contributions:

**NAZ:** Conceptualization, Manual check of data, writing draft.

**TSA and MHI:** Data extraction and analysis of data, Manuscript writing.

**MAH:** Manual check of data, Critical revision of Manuscript.

**FUG:** Data Analysis, Critical revision of Manuscript.

All authors have approved the final version and are accountable for the integrity of the study.
